# Characterisation of prostate cancer lesions in heterozygous *Men1 *mutant mice

**DOI:** 10.1186/1471-2407-10-395

**Published:** 2010-07-27

**Authors:** Christelle Seigne, Sandra Fontanière, Christine Carreira, Jieli Lu, Wei-Ming Tong, Bernard Fontanière, Zhao-Qi Wang, Chang Xian Zhang, Lucien Frappart

**Affiliations:** 1 CNRS UMR5201, Laboratoire de Génétique Moléculaire, Signalisation et Cancer, Centre Léon Bérard, Lyon F-69008, France; 2 International Agency for Research on Cancer, Lyon F-69008, France; 3 Institute of Basic Medical Sciences, Academy of Medical Sciences, Beijing Union Medical College, Beijing-100005, China; 4 Département de Pathologie, Centre Léon Bérard, Lyon F-69008, France; 5 Leibniz Institute for Age Research - Fritz Lipmann Institute e.V., 07745 Jena, Germany; 6 Département de Pathologie, INSERM U590, Centre Léon Bérard, Hôpital Edouard Herriot, HCL, Lyon F-69008, France; 7 The E-Institute of Shanghai, Sino-French Life Science and Genomic Research Center, Jiaotong University, Shanghai, China

## Abstract

**Background:**

Mutations of the *MEN1 *gene predispose to multiple endocrine neoplasia type 1 (MEN1) syndrome. Our group and others have shown that *Men1 *disruption in mice recapitulates MEN1 pathology. Intriguingly, rare lesions in hormone-dependent tissues, such as prostate and mammary glands, were also observed in the *Men1 *mutant mice.

**Methods:**

To study the occurrence of prostate lesions, we followed a male mouse cohort of 47 *Men1*^+/- ^mice and 23 age-matched control littermates, starting at 18 months of age, and analysed the prostate glands from the cohort.

**Results:**

Six *Men1*^+/- ^mice (12.8%) developed prostate cancer, including two adenocarcinomas and four *in situ *carcinomas, while none of the control mice developed cancerous lesions. The expression of menin encoded by the *Men1 *gene was found to be drastically reduced in all carcinomas, and partial LOH of the wild-type *Men1 *allele was detected in three of the five analysed lesions. Using immunostaining for the androgen receptor and p63, a basal epithelial cell marker, we demonstrated that the menin-negative prostate cancer cells did not display p63 expression and that the androgen receptor was expressed but more heterogeneous in these lesions. Furthermore, our data showed that the expression of the cyclin-dependent kinase inhibitor CDKN1B (p27), a *Men1 *target gene known to be inactivated during prostate cell tumorigenesis, was notably decreased in the prostate cancers that developed in the mutant mice.

**Conclusion:**

Our work suggests the possible involvement of *Men1 *inactivation in the tumorigenesis of the prostate gland.

## Background

Mutations of the *MEN1 *gene predispose patients to multiple endocrine neoplasia type 1 (MEN1), characterised by the occurrence of multiple endocrine tumours affecting mainly the parathyroid glands, the endocrine pancreas and the anterior pituitary [1]. Shortly after the identification of the *MEN1 *gene, several nonendocrine tumours were also reported in MEN1 patients, such as lipoma and angioma [2,3]. The *MEN1 *gene encodes a primarily nuclear-localised protein named menin that has been shown to interact with a variety of other proteins [4]. In particular, its interaction with several transcriptional factors and co-factors suggests that menin may act as an adaptor protein involved in the regulation of gene expression. However, little is known about the *in vivo *physiological function of menin and the *in vivo *importance of the aforementioned protein-protein interactions, especially in the endocrine cells commonly affected in MEN1 disease.

By disrupting *Men1 *in mice, we and others have previously shown that, while homozygous *Men1 *mutant mice die embryonically, heterozygous *Men1 *mutant mice largely recapitulate the major endocrine lesions seen in MEN1 pathology [5-8]. However, these mice develop several types of neoplastic lesions that are rarely seen in MEN1 patients, such as high frequency sex-cord stromal cell tumours in 88% of aged male mice and 50% of female mice [6,8]. Intriguingly, we have found three cases (3/36, 8,3%) of breast cancer in heterozygous *Men1 *female mice [6], and Crabtree *et al*. documented one case of prostate cancer developed in their heterozygous *Men1 *mouse cohort [7]. Consistent with these observations, Dreijerink *et al*. have shown that menin interacts physically, in a ligand-dependent manner, with several nuclear receptors, such as the oestrogen receptor (ER) [9] and peroxisome proliferator activated receptor gamma (PPARγ) [10], and that menin acts as a coactivator of nuclear receptor mediated transcription. These studies raise the question of whether *Men1 *inactivation could predispose to the development of hormone-dependent tumours. To investigate the possible link between the development of prostate cancer and *Men1 *disruption, we followed a cohort of aged male heterozygous *Men1 *mutant mice. Several cases of prostate cancer were found in this group, whereas no cases were seen in control animals. Interestingly, the expression of menin was inactivated in these prostate cancers, and partial LOH (loss of heterozygosity) of the wild-type *Men1 *allele was found. Gene expression analyses further revealed that the expression of CDKN1B (cyclin-dependent kinase inhibitor 1B, also called p27), a known menin target gene [11,12], was markedly reduced in the analysed prostatic carcinomas.

## Methods

### Men1 mutant mice

*Men1*^+/- ^mice carrying an inactivated *Men1 *allele were generated as previously described [5]. These mutant mice with mixed C57BL6/129-Sv genetic background give rise to multiple endocrine lesions mimicking the human MEN1 tumour spectra as previously described [6]. Littermate *Men1*^+/- ^and *Men1*^+/+ ^male mice were used for the analyses to limit the bias possibly originating from the mixed genetic background. All animal experiments were conducted in accordance with accepted standards of animal care and were approved by the International Agency for Research on Cancer's Animal Care and Use Committee.

### Genotyping and LOH analysis

PCR genotyping analyses were performed to determine the presence of the wild-type and mutant *Men1 *alleles as previously described [6]. For LOH analysis, menin-immunostained prostate sections were used to locate menin-negative lesions. Genomic DNA was then extracted from corresponding microdissected paraffin-embedded sections as previously described [6]. The wild-type (+) and mutant (-) *Men1 *alleles were first amplified from 1 μg of genomic DNA by PCR, carried out as described for genotyping [6], using the following three primers: 2f0, 5-CTTACCTCTTCTCATGTCTG; 2r0, 5-CTCAGTACATTGCACGGAGA; and tk1.1, 5-GCGTTGCGTGGGGTCAG. Southern blots were then performed by standard methods on these PCR products using a probe for each *Men1 *allele generated by amplification of genomic DNA from a *Men1 *heterozygous mutant mouse with the PCR described above. Autoradiography films were scanned with the EC3 imaging system (UVP Bioimaging Systems), and the intensity of each band was quantified with Visionworks LS software. The ratio between the intensities of the + and - *Men1 *alleles (+/- ratio) was then calculated to evaluate the loss of the wild-type *Men1 *allele.

### Histopathological analyses

Prostate glands and adjacent tissues were collected from wild-type and heterozygous *Men1 *mutant mice, fixed in 4% buffered formalin for at least 24 h at room temperature, and processed for paraffin-embedding, sectioning (3 μm) and haematoxylin and eosin staining. Histopathological examination of prostate samples was performed independently by three pathologists (B Fontanière, L Frappart and WM Tong), including a mouse model specialist (WM Tong), on a blinded basis according to the criteria recommended by the consensus report from the Bar Harbor meeting of the mouse models of human cancer consortium (MMHCC) prostate pathology committee [13]. The WHO Classification of Tumours of the Urinary System and Male Genital Organs (2004) was also used. More than 3 prostate sections were examined for each mouse and lobe origin was distinguished during the scoring process. The final diagnosis was then reached by a common decision.

### Immunostaining analysis

Immunohistochemical (IHC) staining was performed on prostate serial sections as previously described [6], using antibodies against menin (BL342, Bethyl Laboratories, Montgomery, TX, USA, 1:6000), androgen receptor (C-19, Santa Cruz Biotech, Santa Cruz, CA, USA, 1:4000) and CDKN1B (F-8, Santa Cruz Biotech, 1:2000). In all these analyses, a control without primary antibody was systematically included to rule out non-specific staining due to secondary antibody reactivity (data not shown). Immunofluorescence (IF) double staining was performed on paraffin-embedded prostate sections. Briefly, after endogenous peroxidase inactivation, antigen retrieval and antibody diluent treatment (DAKO, Capinteria, CA, USA), sections were incubated overnight with primary antibody against menin (BL342, 1:6000). After secondary antibody incubation (anti-rabbit, Vector Lab, 1:200), the signal was amplified using the ABC Vectastain elite kit (Vector Lab) and the TSA Cyanine 3 kit (PerkinElmer, Waltham, MA, USA, 1/100). Slides were saturated once more in DAKO antibody diluent and then incubated overnight with primary antibodies against p63 (Ab-1, Oncogene Research products, San Diego, CA, USA, 1:2000) or CDKN1B (Santa Cruz Biotech, 1:2000), followed by incubation with secondary antibody (anti-mouse, Vector Lab, 1:200). The signals were revealed by the ABC kit and TSA Cyanine 5 (PerkinElmer, 1/100). Slides were mounted in IF Vectashield DAPI-containing mounting medium (Vector Lab), and the immunofluorescence signal was visualised with a confocal microscope (LEICA TCS SP2).

## Results

### A proportion of aged heterozygous *Men1* mutant mice develop prostate cancer

To determine the effect of *Men1 *inactivation on prostate cancer development in mice, we followed a cohort of 47 male mutant mice (*Men1*^+/-^) and 23 wild-type (*Men1*^+/+^) age-matched littermate mice from 18 to 26 months of age, based on a previous study that showed no prostate cancer in younger mice [6].

**Figure 1:**
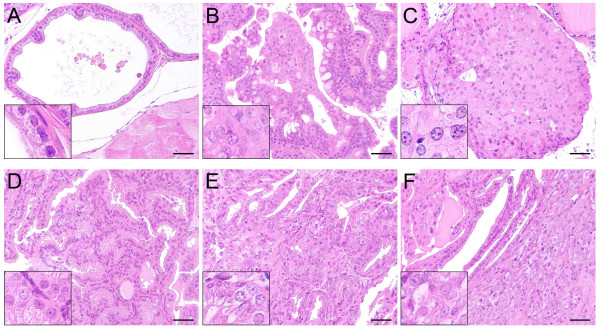
**Precancerous and cancerous lesions are detected in aged male heterozygous *Men1 *mutant mice**. Haematoxylin and eosin staining of a normal lateral prostate lobe from a 24-month-old *Men1*^+/+ ^mouse (A) and precancerous or cancerous lesions observed in prostate glands from heterozygous *Men1 *mutant mice (B-F). (B) mPIN found in the anterior prostate with a signet ring variant: cytoplasmic vacuoles displacing the cell nuclei (21-month-old mouse). (C) *In situ *carcinoma in the dorsal prostate (26-month-old mouse). (D) Adenocarcinoma in the anterior prostate with branching and papillary infolding (23-month-old mouse). (E) Differentiated invasive adenocarcinoma in the lateral prostate (*in situ *component) from a 23-month-old mouse. (F) Invasive component of the same lesion as in (E) showing invasion to the prostatic connective tissue. Insets show a magnified view of a part of the prostate glands. Scale bars, 50 μm.

Histopathologic analysis of the prostate glands from these mice was performed in accordance with the criteria defined by the MMHCC [13]. A comparison between age-matched *Men1 *wild-type and mutant mice revealed notable histological differences, showing a marked increase in cancer incidence in *Men1*^+/- ^prostates (Table 1) and a significantly decreased proportion of mutant mice with normal prostate histology. The results showed 30.4% of *Men1 *wild-type prostates with normal histology while only 6.4% of prostate glands from mutant mice were free of lesions (p = 0.0266, Fisher's two-tailed exact test). Normal prostate tissue presented as a single layer of secretory epithelial cells lined by a well-defined outer basal cell layer, with uniform small nuclei containing inconspicuous or small nucleoli. The epithelial hyperplasia diagnosed in this study appeared as increased numbers of epithelial cells, with or without atypia, associated with increased gland size. The intraluminal proliferation of markedly atypical epithelial cells, with tufted, cribriform or micropapillary growth pattern, was recognised histologically as mouse prostatic intraepithelial neoplasia (mPIN) (Figure 1B). Carcinomas were characterised by frequent mitotic figures, apoptotic debris and cytologic atypia, such as amphophilic cytoplasm, increased nuclear-cytoplasmic ratio, hyperchromasia, prominent and multiple nucleoli, chromatin clumping and pleomorphism (Figure 1C, D, E).

**Table 1:** Morphologic alterations in the prostate glands of male heterozygous *Men1 *mice.

Type of pathology	*Men1*^+/+^	*Men1*^+/-^
Normal prostate	7/23 (30.4%)^a^	3/47 (6.4%)*
Hyperplasia	10/23 (43.5%)	22/47 (46.8%)
mPIN	6/23 (26.1%)	16/47 (34%)
Carcinoma	0/23 (0%)	6/47 (12.8%)

*Men1*^+/+ ^and *Men1*^+/- ^male mice were monitored for cancer development and examined by histology between 18 and 26 months. ^a ^Number of mice of each histological category over the total number of the mice examined. The percentage of mice in each histological category is shown in parentheses. For each mouse, only the most severe type of lesion was taken into account. For this reason, mPINs observed in four mice having carcinoma lesions were not counted in this table. * p = 0.0266 (Fisher's exact test).

Hyperplastic and mPIN lesions, mainly presenting in the lateral and anterior lobes of prostatic glands, were observed with similar frequency in mutant and wild-type aged mice (Table 1, 43.5% in *Men1*^+/+ ^versus 46.8% in *Men1*^+/- ^mice for hyperplasia and 26.1% in *Men1*^+/+ ^versus 34% in *Men1*^+/- ^mice for mPIN), and a few of them were also found in dorsal and ventral lobes. However, the incidence of mPIN in mutant mice was underestimated because four mPIN lesions observed in mutant mice with *in situ *carcinomas were not counted, since only the most severe lesion was taken into account for each mouse. The real incidence of mPIN in *Men1*^+/- ^mice should thus be 42.5%. The occurrence of both hyperplasia and mPIN lesions has already been reported in some wild-type mouse strains [14].

More importantly, our analysis revealed that, of 47 heterozygous *Men1 *mutant mice, six developed prostate cancers (12.8%). No prostate carcinoma was ever found in age-matched *Men1*^+/+ ^littermates (0/23). Among the prostate cancers observed in *Men1*^+/- ^mice, four were identified as *in situ *prostate carcinomas presenting as microscopic lesions (one in dorsal prostate, shown in Figure 1C, two in lateral prostate, and one in anterior prostate), one papillary adenocarcinoma in the anterior prostate (Figure 1D) and one differentiated invasive adenocarcinoma in the lateral prostate (Figure 1E, F), with the latter two visible on gross examination. Invasive tissue was found in epithelial areas lacking an intact basal cell layer, with cancerous cells invading the surrounding stroma (Figure 1F). Among the observed prostatic lesions, none displayed histopathological features typical of neuroendocrine differentiation (solid or sheet-like proliferation of closely spaced oval or spindle cells with scant cytoplasm and hyperchromatic nuclei, with areas of rosette formation) [13].

Taken together, our data revealed the development of prostate carcinomas in a small proportion of aged heterozygous *Men1 *mutant mice.

### Menin expression is inactivated in cancerous lesions mainly through LOH

**Figure 2:**
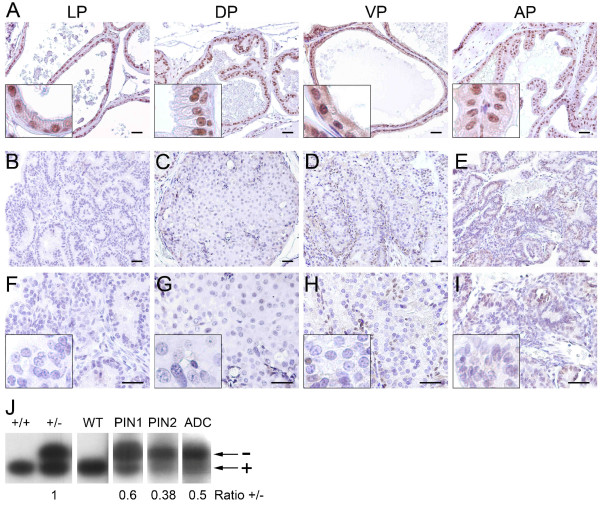
**Menin expression is inactivated in prostate cancers from *Men1*^+/- ^mice**. Microscopic images of prostate glands from *Men1*^+/+ ^(A) and *Men1*^+/- ^mice (B-I) subjected to menin detection by IHC. Menin is widely expressed in the nuclei of prostate epithelial cells in the lateral (LP), dorsal (DP), ventral (VP) and anterior (AP) prostate from a 21-month-old *Men1*^+/+ ^mouse (A), but is completely inactivated in two of four tested mPINs and in all six prostatic cancerous lesions in *Men1*^+/- ^mice. Four representative types of lesions are shown: mPIN from a 21-month-old *Men1*^+/- ^mouse (B, F), an *in situ *prostate carcinoma from a 26-month-old mouse (C, G), a well-differentiated adenocarcinoma from a 23-month-old mouse (D, H) and a papillary adenocarcinoma from a 23-month-old mouse (E, I). Panels F-I are two-fold magnifications of the upper panels (B-E). Insets show an amplified view of a part of the prostate glands. Scale bars, 50 μm. (J) Representative results from two independent LOH analyses of prostatic lesions in *Men1*^+/- ^mice. Semi-quantitative amplification of *Men1 *wild-type (+) and mutant (-) alleles with PCR was performed on DNA samples extracted from microdissected paraffin-embedded sections from *Men1*^+/+ ^normal prostate (WT) and prostate lesions from *Men1*^+/- ^mice, including two mPIN (PIN1 and PIN2) and one adenocarcinoma (ADC). Tail DNA from wild-type (+/+) and heterozygous (+/-) *Men1 *mice were used as controls. The intensity of both alleles (+ and -) was quantified and used to calculate the +/- ratio, which was compared with the +/- ratio obtained from the controls.

**Figure 3:**
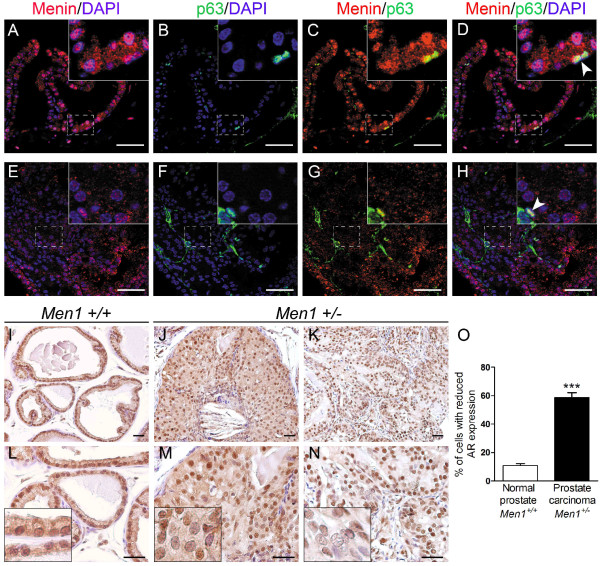
**Prostate cancers from *Men1*^+/- ^mice do not express p63 and display heterogeneous AR expression**. (A-H) Double IF staining using antibodies against menin (red) and p63 (green) performed on a normal prostate from a 24-month-old *Men1*^+/+ ^mouse (A-D, n = 2) and an adenocarcinoma found in a *Men1*^+/- ^mouse of the same age (E-H, n = 2). DAPI stains cell nuclei. Note that normal p63-expressing basal cells scattered within the lesion remain menin-positive (shown by white arrowheads). Boxed areas are magnified in the insets. Scale bars, 50 μm. (I-N) IHC using antibody against AR was performed on prostate tissues from *Men1*^+/+ ^(I, L, n = 4) and *Men1*^+/- ^(J, M and K, N, n = 4) mice. AR expression is present but more heterogeneous in an *in situ *prostate carcinoma (J, M, 26-month-old mouse) and an adenocarcinoma (K, N, 23-month-old mouse) from *Men1*^+/- ^mice, with some cells showing greatly reduced AR expression compared to the control. Panels L-N are two-fold magnifications of the upper panels (I-K). Insets show an amplified view of a part of the prostate glands. (O) Prostatic epithelial nuclei showing absent or very low AR expression were counted in six random microscopic fields in normal prostate glands from *Men1*^+/+ ^mice (n = 3) and carcinomas observed in *Men1*^+/- ^prostate glands (n = 4), stained with an anti-AR antibody. The results are expressed as a percentage of total prostatic epithelial cells (250 nuclei counts). Values are means ± standard error of the mean. *** Significant difference from normal *Men1*^+/+ ^prostate with a two-tailed Student's *t*-test (*P *< 0.0001).

Menin expression was assessed in the different prostatic lobes from *Men1 *wild-type mice using either IHC for menin or double IF with antibodies against menin and p63, a basal epithelial cell marker. IHC analysis revealed that menin expression, which is mainly nuclear but can also be detected in the cytoplasm, was found in all prostatic lobes from *Men1 *wild-type mice in both luminal and basal prostatic epithelial cells (Figure 2A) but not in interstitial cells. Double IF staining of menin and p63 confirmed this result (Figure 3A-D). To evaluate the association between the occurrence of prostatic lesions in heterozygous *Men1 *mice and inactivation of the *Men1 *gene, we examined menin expression by IHC in the corresponding lesions. We found that menin expression was readily detectable by IHC in all four tested mPIN lesions from WT mice (data not shown), but clearly reduced in two of four mPIN lesions from mutant mice (Figure 2B, F). The data suggest that, although a large proportion of mPINs observed in mutant mice occur independently of *Men1 *inactivation, as those seen in wild-type mice, some may still be related to menin loss. In contrast to mPIN lesions, no menin expression was detectable in the six prostate cancers found in *Men1*^+/- ^mice (Figure 2C-E and 2G-I), suggesting that prostate cancer in these mice was likely caused by menin inactivation, rather than the secondary effects of MEN1 pathology, such as gene expression and/or hormone disturbance in endocrine tissues. In two of the prostate cancers shown in Figure 2 (panels D, H and E, I), a small number of cells remain positive for menin expression. We reasoned that these cells were in fact normal basal epithelial cells that remained in cancer lesions, which was confirmed by further analyses (see below).

To further study the related mechanisms leading to menin inactivation, we tested the presence of LOH in mPIN from *Men1*^+/- ^mice displaying reduced menin expression and in menin-negative carcinoma lesions detected by IHC analysis. To this end, the tested tissues were scraped from the corresponding paraffin-embedded slides, and genomic DNA was extracted from each lesion. Genotyping analysis demonstrated that partial LOH was indeed found in three of five analysed precancerous and cancerous lesions (Figure 2J). The quantification of amplified products corresponding to *Men1^+ ^*and *Men1*^- ^alleles from the tail of a *Men1*^+/- ^mouse was used as a control, and an identical intensity of both alleles was achieved, thus giving a +/- ratio equal to one. The intensity of the wild-type allele (+), but not the mutant allele (-), was reduced in two mPINs (PIN1 and 2, both showing reduction of menin expression by IHC analysis) and in an adenocarcinoma lesion (ADC) from *Men1*^+/- ^mice, as demonstrated by the lower +/- ratios for these lesions when compared with the control (Figure 2J). We assume that the residual wild-type allele, amplified from the tested lesions, was derived mainly from the remaining normal basal cells within the lesions, which were shown to retain menin expression. The result suggests that LOH of the remaining wild-type *Men1 *allele may be one of the mechanisms leading to menin inactivation in the prostate cancers developed in this model, although we cannot exclude that other mechanisms may also be involved.

### The prostate cancers developed in heterozygous *Men1 *mutant mice do not express p63 and display heterogeneous AR expression

Although a loss of menin expression and a partial LOH were found in some mPINs from *Men1 *mutant mice, these occurred at similar frequencies in wild-type and mutant mice. The present study therefore focused on the analysis of the prostate cancers arising in *Men1 *mutant mice. To further characterise the observed cancer lesions, expression of the basal epithelial marker p63 was analysed using double IF staining, since the diagnosis of cancerous lesions can often be based on the disruption of the basal cell layer indicated by the absence or greatly reduced number of p63-positive basal cells [15]. Menin-negative cancerous cells were negatively stained with basal cell marker p63 (Figure 3E-H). In addition, normal p63-positive basal cells, which retained menin expression, were greatly reduced in number and scattered in the cancerous lesions, showing a disruption of the basal cell layer (Figure 3H, white arrowhead). The data were thus consistent with our histopathologic findings and indicated that menin inactivation did not occur in basal cells, confirming what we had speculated on the basis of the results obtained by menin IHC.

Prostatic cells express the androgen receptor (AR) and respond to the androgen pathway that regulates prostate growth, apoptosis and differentiation. Nearly 70% of clinical prostate cancer cases are AR positive. It is generally considered that AR expression in prostate cancer cells reflects not only the differentiation status of the cells, but also their sensitivity to anti-AR treatment. We therefore evaluated AR expression using IHC in different prostate tissue samples. Normal prostate tissue from *Men1*^+/+ ^mice showed strong AR nuclear staining in both luminal and basal epithelial cells, as well as in many stromal cells (Figure 3I, L), as previously described in the literature [16-18]. AR was clearly expressed in an *in situ *carcinoma (Figure 3J, M) and an adenocarcinoma (Figure 3K, N) and showed a heterogeneous expression pattern in these lesions in comparison with the normal control prostate (Figure 3I, L), with some cells showing absent or very low AR staining. Quantitation of epithelial cell nuclei displaying either no AR expression or very low staining revealed a significant increase in the number of cells with altered AR expression in prostate carcinomas from *Men1 *mutant mice compared with normal *Men1*^+/+ ^prostate glands (Figure 3O).

The data demonstrated the disruption of the basal cell layer in these lesions and suggested a possible association between menin inactivation and the deregulation of AR expression.

### The reduced expression of CDKN1B was evidenced in prostatic carcinomas

**Figure 4:**
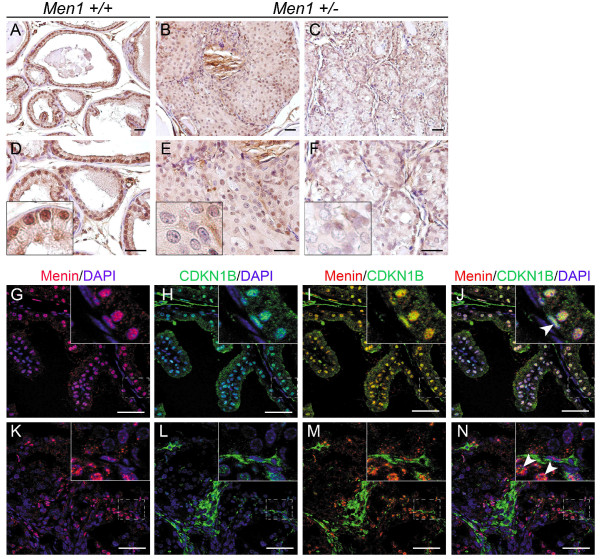
**Reduced CDKN1B expression in prostate cancers from *Men1*^+/- ^mice**. (A-F) IHC was performed on paraffin-embedded sections of prostate tissues from *Men1*^+/+ ^(A, D, n = 4) and *Men1*^+/- ^(B, E and C, F, n = 4) mice using an antibody against CDKN1B. Note that in the normal prostate from aged *Men1*^+/+ ^mice (26-month-old), CDKN1B is expressed in all prostatic epithelial cells, but not in all stromal cells (A, D). CDKN1B expression is reduced in an *in situ *carcinoma (B, E, 26-month-old mouse) and an adenocarcinoma (C, F, 23-month-old mouse) from *Men1*^+/- ^mice when compared with the wild-type prostate. Panels D-F are two-fold magnifications of the upper panels (A-C). Insets show an amplified view of a part of the prostate glands. (G-N) Menin (red) and CDKN1B (green) expressions examined by double IF staining in prostate glands from a 21-month-old *Men1 *wild-type mouse (G-J) and an adenocarcinoma from a 23-month-old *Men1*^+/- ^mouse (K-N). DAPI stains cell nuclei. Note that menin and CDKN1B expressions are co-localised in normal prostate epithelium, whereas both disappear in the cancerous lesions. Arrowheads show that basal cells remain both menin and CDKN1B positive in cancerous lesions. Boxed areas are magnified in the insets. Scale bars, 50 μm.

CDKN1B, an inhibitor of cyclin-dependent kinases, is a transcriptional target of the menin protein [11,12]. The involvement of its downregulation in the tumorigenesis of prostate cells has also been well documented in different mouse models and in prostate cancer patients [19,20]. We therefore analysed the expression of CDKN1B using IHC in serial sections of prostate carcinomas from *Men1*^+/- ^mice. Strong, predominantly nuclear CDKN1B expression was detected in virtually all luminal and basal *Men1*^+/+ ^prostate cells (Figure 4A, D), consistent with previous reports [21,22]. CDKN1B nuclear expression was clearly reduced in an *in situ *carcinoma (Figure 4B, E) and almost absent in an adenocarcinoma (Figure 4C, F) from *Men1*^+/- ^mice. Furthermore, double IF staining with menin and CDKN1B antibodies showed that the reduced expression of CDKN1B in prostate carcinomas correlated well with the loss of menin expression (Figure 4K-N), indicating that CDKN1B expression is indeed down-regulated in menin-negative prostatic cells.

## Discussion

In the current study, analysis of a cohort of aged male *Men1*^+/- ^mice showed a significant reduction in the number of mutant mice with normal prostate glands due to the occurrence of prostate lesions in these mice. Interestingly, six mutant mice developed prostate cancer, which was not observed in the age-matched wild-type littermates. The prostate lesions found in heterozygous *Men1 *mutant mice were characterised by a slow cancer development process, which ranged from *in situ *carcinoma to invasive adenocarcinoma. This cancer development pattern in aged male heterozygous *Men1 *mice is similar to other non SV40-TAg GEM prostate cancer models, likely reflecting the relative late and slow features of prostate cancer development in men. Although one prostate cancer case was previously documented in an independent heterozygous *Men1 *mutant mouse cohort [7], to our knowledge, the present study is the first systematic evaluation and characterisation of prostate cancer related to the inactivation of the *Men1 *gene.

Importantly, the menin protein was undetectable in cancerous cells, and partial LOH was found in three of five pre-and/or cancerous lesions from *Men1*^+/- ^mice. Menin loss in these mutant mice may indicate a close relationship between *Men1 *inactivation and the development of prostate cancer, suggesting that the *Men1 *gene may possess oncosuppressive activity and be involved in the control of cell proliferation in prostate epithelial cells. However, we noticed that the occurrence of prostate cancer in these mice can be seen only in aged mice with a low frequency, implying that *Men1 *inactivation may confer a relatively minor predisposition to prostate cancer development compared with the endocrine tissues affected in MEN1 pathology and that other factors may be involved in the development of this pathology. Interestingly, these results are similar to the findings with mice carrying either one mutated *Pten *or the *Nkx3.1 *allele. Male heterozygous *Pten *mice develop mPIN at ages older than 9 months [19], whereas *Nxk3.1 *mutant mice show only hyperplastic or dysplastic prostatic epithelium [23]. However, the double mutant *Pten*^+/-^: *Nxk3.1*^+/- ^mice display high grade PIN/early carcinoma lesions at a high frequency [24,25]. Both genes have been found either mutated or down-regulated in human prostate cancer [19,26]. The results of the present study suggest that the *Men1 *gene could be among the rare known tumour suppressors whose disruption leads to the development of prostate cancer in mice, albeit at low incidence and with a slow progression rate.

Intriguingly, a recent study reported that gain at the *MEN1 *locus was detected in a substantial proportion of human metastatic prostate cancers, and a trend toward increased menin expression in human prostate cancers and metastatic tissues was suggested by a meta-analysis of *MEN1 *expression data in prostate cancer [27]. Similarly, Imachi *et al*. reported that menin expression in breast cancers could be used as a prognostic factor of worse outcome [28]. Knowing the multifaceted role played by the *MEN1 *gene, menin may play an oncogenic role under certain circumstances in these tissues, particularly in recurring and aggressive cancers, in similarity to the well-known dual role played by the TGF-β pathway, whose several effectors are the protein partners of menin, in the process of tumourigenesis. However, further clinical studies are needed to validate these observations, as mentioned by the authors.

Our results suggest that the study of the potential link between menin and AR expression or activity in prostatic cells would be of great interest, as the latter is deregulated in the prostate cancers found in *Men1 *mutant mice. Generally considered a co-regulator of transcription, menin interacts physically and functionally with several nuclear receptors, such as ERα [9] and PPARγ [10], playing the role of a transcriptional coactivator via its LXXLL motif. Although a physical interaction between menin and AR has not been reported yet, the existence of cross-talk between menin and the AR pathways is possible. Indeed, menin and AR share common partners like Smad3, a downstream effector of the TGF-β signalling pathway, and β-catenin, an effector of WNT pathway [4,29], but also common target genes important for cell cycle control, such as *CDKN1B *and *cyclins D *[4,11,12,30,31]. The observation of deregulated AR expression in the cancerous lesions found in *Men1 *mutant mice suggests that menin inactivation and subsequent AR deregulation in these cells may lead to the disturbance of the AR pathway, which could in turn promote the progression to cancer.

It is worth mentioning that the mutant mice enrolled in this study developed Leydig cell tumours with high frequency, which could suggest that the occurrence of these tumours plays a role in the tumorigenesis of prostate cells. However, the data from previously published mouse models of Leydig cell tumours did not offer any evidence supporting this hypothesis, as none reported prostate lesions [32-36]. Furthermore, AR signalling seems to play a growth suppressing function in the initial stage of tumour development [30]. Nevertheless, it will be interesting in the future to investigate the possible interplay between the development of Leydig cell tumours and the occurrence of prostate cancer.

Prior studies have proposed that the growth inhibition function of AR signalling in normal prostatic luminal cells is mediated by its induction of CDKN1B expression [30]. This indeed correlates with the downregulation of CDKN1B observed in prostate carcinomas developed in our *Men1 *mutant mice. CDKN1B, which is closely related to the tumorigenesis of prostate cancers as described above, is among the transcriptional targets of menin. The mouse models with *CDKN1B *disruption in combination with other genetic factors, such as *Pten *and *Nkx3.1*, have been shown to develop a range of prostate cancers, although *CDKN1B *inactivation alone displays no obvious neoplastic prostate lesions [19,25]. The downregulation of CDKN1B found in prostate lesions in *Men1 *mutant mice suggests the *in vivo *importance of CDKN1B inactivation in the tumorigenesis related to *Men1 *inactivation, similar to observations in other mouse *Men1 *tumour models [12,37-39]. Furthermore, it would be interesting to clarify the possible relation between menin and other factors involved in CDKN1B regulation in the prostate, such as Pten and Nkx3.1, in the future.

## Conclusion

The presented data suggest the possible involvement of *Men1 *inactivation in the tumorigenesis of prostatic cells in mice. Recent extensive genetic studies highlighted the difficulties in identifying genes involved in the tumorigenesis of prostate cells [40], indicating that prostate cancer could be a genetically complex disease with multiple predisposing factors affecting initiation, progression, and outcome of the disease. It would thus be interesting to investigate the eventual oncosuppressive role of the *Men1 *gene in prostatic cells in humans in the future. The current study also suggests that both altered AR and CDKN1B expression may be among the factors participating in or facilitating the tumorigenesis of prostatic cells due to *Men1 *inactivation.

## Competing interests

The authors declare that they have no competing interests.

## Authors' contributions

CS conducted most of the experiments, analysed data and prepared figures; SF maintained the mouse colony and collected tissues; CC performed experiments, in particular histological analyses; JL provided experimental advice and assistance; WMT, BF and LF performed the histopathological analyses; ZQW supervised the experiments and helped preparing the manuscript; and CXZ and LF conceived the study, supervised the overall project, analysed data and wrote the manuscript. All authors read and approved the final manuscript.

## Pre-publication history

The pre-publication history for this paper can be accessed here:

http://www.biomedcentral.com/1471-2407/10/395/prepub
